# The accumulation of myeloid‐derived suppressor cells participates in abdominal infection‐induced tumor progression through the PD‐L1/PD‐1 axis

**DOI:** 10.1002/1878-0261.13767

**Published:** 2025-01-21

**Authors:** Yiding Wang, Ting Guo, Xiaofang Xing, Xijuan Liu, Xuejun Gan, Yingai Li, Yan Liu, Fei Shan, Zhouqiao Wu, Jiafu Ji, Ziyu Li

**Affiliations:** ^1^ Department of Gastrointestinal Cancer Translational Research, Key Laboratory of Carcinogenesis and Translational Research (Ministry of Education) Peking University Cancer Hospital & Institute Beijing China; ^2^ Department of Gastrointestinal Cancer Center Peking University Cancer Hospital & Institute Beijing China; ^3^ Department of Central Laboratory, Key Laboratory of Carcinogenesis and Translational Research (Ministry of Education/Beijing) Peking University Cancer Hospital & Institute Beijing China

**Keywords:** gastric cancer, intra‐abdominal infectious complications, MDSC, PD‐1, tumor microenvironment

## Abstract

Gastric cancer (GC) is the third leading cause of cancer‐related deaths worldwide, with gastrectomy being the primary treatment option. Sepsis, a systemic inflammatory response to infection, may influence tumor growth by creating an immunosuppressive environment conducive to cancer cell proliferation and metastasis. Here, the effect of abdominal infection on tumor growth and metastasis was investigated through the implementation of a peritoneal metastasis model and a subcutaneous tumor model. In a murine model induced by cecal ligation and puncture (CLP) to simulate the effects of sepsis, we observed significant immune dysregulation, including T‐cell exhaustion and the release of myeloid‐derived suppressor cells (MDSCs). This immune alteration was associated with increased programmed cell death protein 1 (PD‐1) expression on T cells and programmed cell death 1 ligand 1 (PD‐L1) expression on MDSCs within the tumor microenvironment, fostering an immune‐suppressive environment. Polymorphonuclear MDSCs (PMN‐MDSCs) expressing elevated PD‐L1 after sepsis demonstrated more substantial suppressive effects on T‐cell proliferation than controls. Treatment with anti‐PD‐1 monoclonal antibodies successfully restored T‐cell function, reduced mortality, and decreased metastasis in CLP mice. These findings emphasize the impact of sepsis on tumor progression and suggest targeting the PD‐1/PD‐L1 axis as a potential therapeutic strategy for managing immune dysfunction in patients with cancer.

## Graphical abstract

This study explores how sepsis affects GC progression by creating an immunosuppressive environment. Our findings reveal that sepsis promotes immune dysregulation, enhancing tumor growth and metastasis. Targeting the PD‐1/PD‐L1 pathway with monoclonal antibodies shows potential for restoring immune function and improving outcomes in cancer patients affected by sepsis.

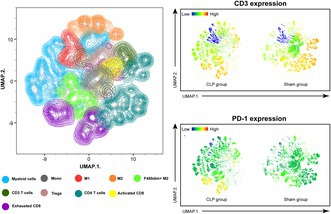

**Abbreviations**
ALB: albumin
ALT: alanine aminotransferase
ANOVA: analysis of variance
AST: aspartate aminotransferase
CK: creatine kinase
CLP: cecal ligation and puncture
DBIL: direct bilirubin
GC: gastric cancer
IFs: inflammatory factors
LDH: lactate dehydrogenase
MDSCs: myeloid‐derived suppressor cells
MFI: mean fluorescence intensity
M‐MDSC: monocytic MDSCs
MMPs: metalloproteinases
PBS: phosphate‐buffered saline
PD‐1: programmed cell death protein 1
PD‐L1: programmed cell death 1 ligand 1
PMN‐MDSCs: polymorphonuclear MDSCs
SD: standard deviation
SEM: standard error of mean
UMAP: Uniform Manifold Approximation and Projection

## 1 Introduction

The incidence of gastric cancer (GC) ranks fifth globally, making it the third leading cause of cancer‐related mortality. In 2020 alone, over 1 million individuals were diagnosed with GC worldwide, with approximately three‐quarters succumbing to the disease within a few years [1, 2]. Surgical resection has been the primary treatment method since Theodor Billroth conducted the world's first successful gastrectomy. Since that milestone, concerns about PIICs have been paramount among surgeons and patients alike [3]. Recent studies have revealed that the incidence of PIICs, such as anastomotic leakage and intra‐abdominal abscess, ranges from 3.3% to 10.6% [4, 5]. Currently, PIICs stand as the predominant cause of perioperative death for GC patients, and several studies have consistently demonstrated a significant association between PIICs and inferior survival outcomes in individuals diagnosed with GC [4, 6, 7].

The intricate immune regulation induced by abdominal infection plays a pivotal role in remodeling the abdominal microenvironment. During the initial stages of anastomotic healing, surgical interventions elicit physiological inflammatory responses, followed by localized infiltration of inflammatory cells that release various inflammatory factors (IFs), including cytokines and metalloproteinases (MMPs), which are crucial for the early phases of healing [8]. However, prolonged polymicrobial abdominal infections result in the dysregulation of the immune system, characterized by lymphopenia and impaired immune function resulting from a loss of B cells and T cells via apoptosis [9]. The immune checkpoint receptor, known as programmed cell death 1 (PD‐1 or CD279), is acknowledged for its critical role in regulating the quantity and functional activity of T cells [10]. Multiple studies have documented elevated PD‐1 expression in lymphocytes following abdominal infections [11, 12, 13, 14]. For instance, Kubo et al. [14] conducted a study involving 30 patients with PIICs, revealing an upregulation of PD‐1 expression in CD4^+^ T cells. The subsequent research further demonstrated an enhancement of PD‐1 expression on peripheral T cells [15], as well as an increased presence of programmed cell death 1 ligand (PD‐L1 or CD274) on spleen B cells and monocytes in a cecal ligation and puncture (CLP) model, which is widely used to mimic the abdominal infection and sepsis [16].

PD‐L1 expression on myeloid‐derived suppressor cells (MDSCs) has been observed across various pathological conditions, reflecting their role in immune regulation. Originating from the bone marrow, MDSCs can be broadly categorized into two groups: polymorphonuclear (PMN‐MDSC) and monocytic (M‐MDSC), which employ different mechanisms to suppress immune responses [17]. Their presence fosters metastatic niche formation by inhibiting anti‐tumor immunity and orchestrating paracrine signaling that fuels tumor progression [18]. Clinical studies underscore MDSCs' association with gastrointestinal tumor aggressiveness and metastasis [19, 20, 21]. Particularly in abdominal infection, MDSCs surge post‐abdominal infections due to impaired myeloid cell differentiation, perpetuating immune suppression via mechanisms like T‐cell apoptosis induction through l‐arginine depletion or PD‐L1 upregulation [22]. MDSC subsets, polymorphonuclear and monocytic, exert distinct immunosuppressive strategies. However, the consequences of abdominal infections on tumor dynamics and the immune microenvironment remain less elucidated.

The primary objective of this study is to explore the potential impact of abdominal infection on tumor recurrence and metastasis through its influence on the immune microenvironment. To investigate this, we implemented the CLP model of intermediate severity. Our findings demonstrated that abdominal cavity infection can create an ecological niche favorable to the proliferation of tumor cells. Noticeably, we observed a substantial accumulation MDSCs in the tumor microenvironment post‐abdominal infection. This pronounced accumulation poses a potential impediment to the proliferation and cytotoxic functions of T cells, orchestrated through the PD‐L1/PD‐1 axis.

## 2 Materials and methods

### 2.1 Flow cytometry

Antibodies to surface and intracellular markers were used to phenotype 11 leukocyte subsets in tumor to generate a 16‐color flow cytometry panel. After blocking of Fc receptors, single cell suspensions from tumor or spleen were incubated with the directly conjugated mouse specific monoclonal antibodies for 30 min at 4 °C in the dark. After washing, Cells were acquired in the BD FACSCelesta flow cytometer and analyzed by bd facsdiva (Version 8.0.1; BD, San Jose, CA, USA) and flowjo (version 10.8.2; BD) software. Antibodies listed in Table S1 were used.

### 2.2 *In vivo* xenograft mouse model

Animal experiments were conducted in strict accordance with institutional guidelines and were approved by the Animal Ethics Committee at Peking University Cancer Hospital (Number: EAEC 2022‐04). Male inbred strain 615 mice (6‐week old, 18–20 g) were procured from the Tianjin Institute of Hematology. Prior to the experiments, all animals were acclimatized to a 12‐h light–dark cycle for 1 week in a temperature‐ and humidity‐controlled environment. 1 × 10^5^ MFC cells were injected subcutaneously into the right flanks of 615 male mice. Tumor growth was monitored every 3 days by measuring the width and length of the tumors with calipers. The volumes of tumors were calculated using the following formula: *V* = (*L* × *W* ^2^) × 0.5 (*L* represents the length and *W* represents the width of each tumor). For the metastatic model, MFC cells (5 × 10^6^ cells/300 μL volume per mouse) were injected into the 615 mice via tail vein (*n* = 10). A week later, mice were randomly assigned to undergo either CLP surgery or a sham operation. CLP surgery was performed as previously described [23]. Briefly, mice were anesthetized via intraperitoneal injection of pentobarbital (50 mg·kg^−1^; Kyoritsu Seiyaku Corporation, Tokyo, Japan). The cecum was ligated 1 cm from the distal pole using a 4‐0 thread, followed by a puncture with a 22‐gauge needle midway between the ligation and cecum tip to induce polymicrobial peritonitis. In the sham group, a similar procedure was conducted on mice, but without ligation and puncture of the cecum. Throughout the experiment, all mice had *ad libitum* access to food and water and were closely monitored at least three times daily for 7 days post‐surgery. Simultaneously, the mice were treated with PD‐1 antibody 10 mg·kg^−1^ by intraperitoneal injection every 3 days. Three weeks after the injection of GC cells, all the mice were sacrificed and the samples were collected. Bouin's solution was injected from the main bronchi to fix the lung tissues.

### 2.3 Measurement of organ injury markers

On days 1, 3, 5, 7, 14, and 21 post‐surgery, whole blood samples were collected from mice. The blood was then centrifuged at 3800 ***g*** for 15 min at room temperature to obtain serum for the measurement of creatine kinase (CK) and its isoenzymes, alanine aminotransferase (ALT), aspartate aminotransferase (AST), and lactate dehydrogenase (LDH). Detection kits for CK, ALT, AST, and LDH were obtained from Roche (Basel, Switzerland) and Rayto (Shenzhen, Guangdong, China). Organ injury markers were measured using an automatic biochemistry analyzer (Chemray 240; Rayto, Shenzhen, China).

### 2.4 Isolation of MDSCs

The isolation of MDSCs was performed as described before [24]. The subcutaneous tumor tissues underwent thorough washing with ice‐cold phosphate‐buffered saline (PBS) and were then minced into 1 mm^3^ fragments using scalpels. The fragments were digested into single cells using the Tumor Dissociation Kit (Miltenyi Biotec, San Diego, CA, USA). The red blood cells (RBCs) were lysed with an RBC lysis buffer (BD Biosciences, San Jose, CA, USA). The cells were then purified using a magnetically assisted MDSC isolation kit (Miltenyi Biotec, San Diego, CA, USA) according to the manufacturer's instructions to obtain high‐purity MDSCs. Briefly, the cells pellet was resuspended in running buffer, and the FcR blocking reagent was added for 10 min at 4 °C to block the Fc receptor.

Anti‐Ly6G‐biotin was added to the cells and incubated for 10 min at 4 °C, followed by washing with running buffer and incubation with anti‐biotin microbeads for 15 min at 4 °C. After further washing, cells were resuspended and separated using an LS column in a MACS Separator's magnetic field (Miltenyi Biotec). The column was rinsed with running buffer, and unlabeled Ly6G^−^ cells were collected. Then, the column was removed to isolate magnetically labeled Ly6G^+^ cells (PMN‐MDSC). For M‐MDSC isolation, unlabeled Ly6G^−^ cells were incubated with anti‐Gr‐1‐Biotin and streptavidin microbeads, and magnetically labeled Gr‐1^+^Ly6G^−^ cells were isolated using an MS column.

### 2.5 Co‐culture of PMN‐MDSC and T cells

The suppression of T‐cell activation by PMN‐MDSCs was assessed following previously established methods [25]. In this study, T cells were labeled with CFSE (Dojindo Laboratories, Kumamoto, Japan) at 37 °C for 15 min, shielded from light. Afterwards, a complete culture medium was added in equal volume, and the system was incubated at 37 °C for 5 min. The cells were then pelleted, and the supernatant was discarded. The labeled cells were plated into 96‐well plates alongside PMN‐MDSCs at varying ratios (5 × 10^4^ T cells/well, PMN‐MDSC = 1/1, 1/2, 1/3, 1/6 T cells/well). Plates were stimulated with mouse T activator CD3/CD28 microbeads (Biolegend, San Diego, CA, USA) for 72 h at 37 °C. Finally, flow cytometry was used to determine the proportions of proliferating T cells.

### 2.6 Statistical analysis

All statistical analyses were performed utilizing graphpad prism 8.0 (GraphPad Software, San Diego, CA, USA), and all data were expressed as mean values ± standard deviation (SD). For normal distributed data, Student's *t*‐test was applied to compare the difference between two groups or one‐way analysis of variance (ANOVA) test was applied to compare the difference among three or more groups. The survival curves were drawn by Kaplan–Meier analysis and the log‐rank test was used to compare the survival differences. Univariate and multivariate Cox proportional hazards model was utilized to evaluate the effects of clinical parameters on survival. Differences were defined as significant: *P < 0.05; **P < 0.01; ***P < 0.001.

## 3 Results

### 3.1 Sepsis induced lymphopenia and neutrophilia in peripheral blood

**Fig. 1:**
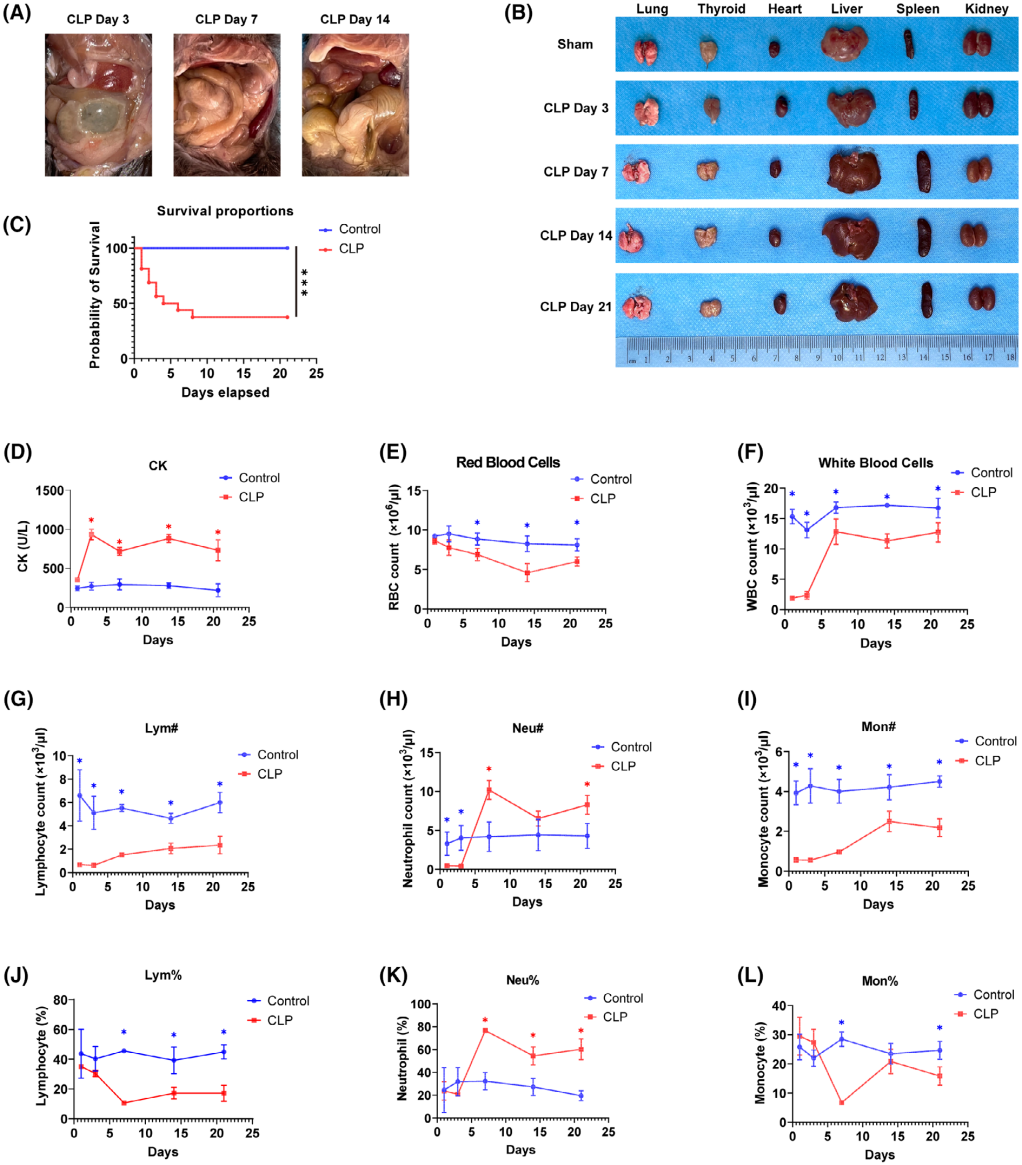
Postoperative abdominal infection, organ damage, and peripheral blood immune status. (A) On the 3rd day after CLP surgery, there was increased abdominal effusion and gradual tissue adhesion; on the 7th day after CLP surgery, the ligated cecum formed an abscess; the abscess persisted on the 14th day after CLP surgery. (B) Splenomegaly appeared on the 7th day after CLP surgery. (C) Survival rate after surgery from day 1 to day 21. Student's *t*‐test, *n* = 15 for each group. (D) Creatine kinase was significantly higher in the CLP group than in Sham group. (E, F) Trends in the numbers of RBCs and WBCs in peripheral blood. Student's *t*‐test. (G–I) Trends in the numbers of lymphocytes, neutrophils, and monocytes in peripheral blood. Student's *t*‐test. (J–L) Trends in the percentages of lymphocytes, neutrophils, and monocytes in peripheral blood. Data from each figure are summarized from three independent time‐course experiments; data are represented as mean ± SD; Student's *t*‐test. *P < 0.05, ***P < 0.001 (compared with the control group). CLP, cecal ligation and puncture; Lym, lymphocyte; Mon, monocyte; Neu, neutrophil; RBC, red blood cell; WBC, white blood cell.

Cecal ligation and puncture is the most widely utilized mouse model for studying the pathogenesis of infection and closely mimics CLP‐induced sepsis observed in patients [26]. CLP surgery involves ligating and puncturing the cecum. Infection signs manifest from the seventh day, including ascites increase by day 3, cecal adhesion, abscess formation by day 7, and spleen enlargement (Fig. 1A,B). The survival data were graphically represented through a Kaplan–Meier survival curve (Fig. 1C). Most CLP mice succumbed within the initial 72 h, with only 37.5% enduring to the seventh day post‐procedure. In stark contrast, the sham‐operated group exhibited a complete survival rate throughout the observation period. Biomarkers (CK, ALT, AST, DBIL, ALB, LDH) assessed organ damage post‐surgery. Elevated CK signifies cardiac injury, while raised ALT and AST imply liver damage [27, 28]. Higher LDH levels suggest tissue injury and hypoxia [29] (Fig. 1D, Fig. S1A–E). These findings validate the successful CLP model establishment, facilitating abdominal inflammation study.

Blood routine tests show that the total white blood cell and red blood cell counts have remained lower than those in the sham group after abdominal infection (Fig. 1E,F). The counts of lymphocytes and monocytes decreased significantly on the first day after infection, and this trend persisted until day 21 (Fig. 1G–I). The count of neutrophils decreased on the first and third days after infection and significantly increased from the seventh day onwards (Fig. 1H). Additionally, from the first day onwards, the percentage of lymphocytes continued to decrease significantly. In contrast, the percentage of neutrophils continued to increase (Fig. 1J–L). These results indicate that inducing abdominal infection in the CLP model leads to lymphopenia and neutrophilia.

### 3.2 Abdominal infection promotes GC tumor growth and metastasis

**Fig. 2:**
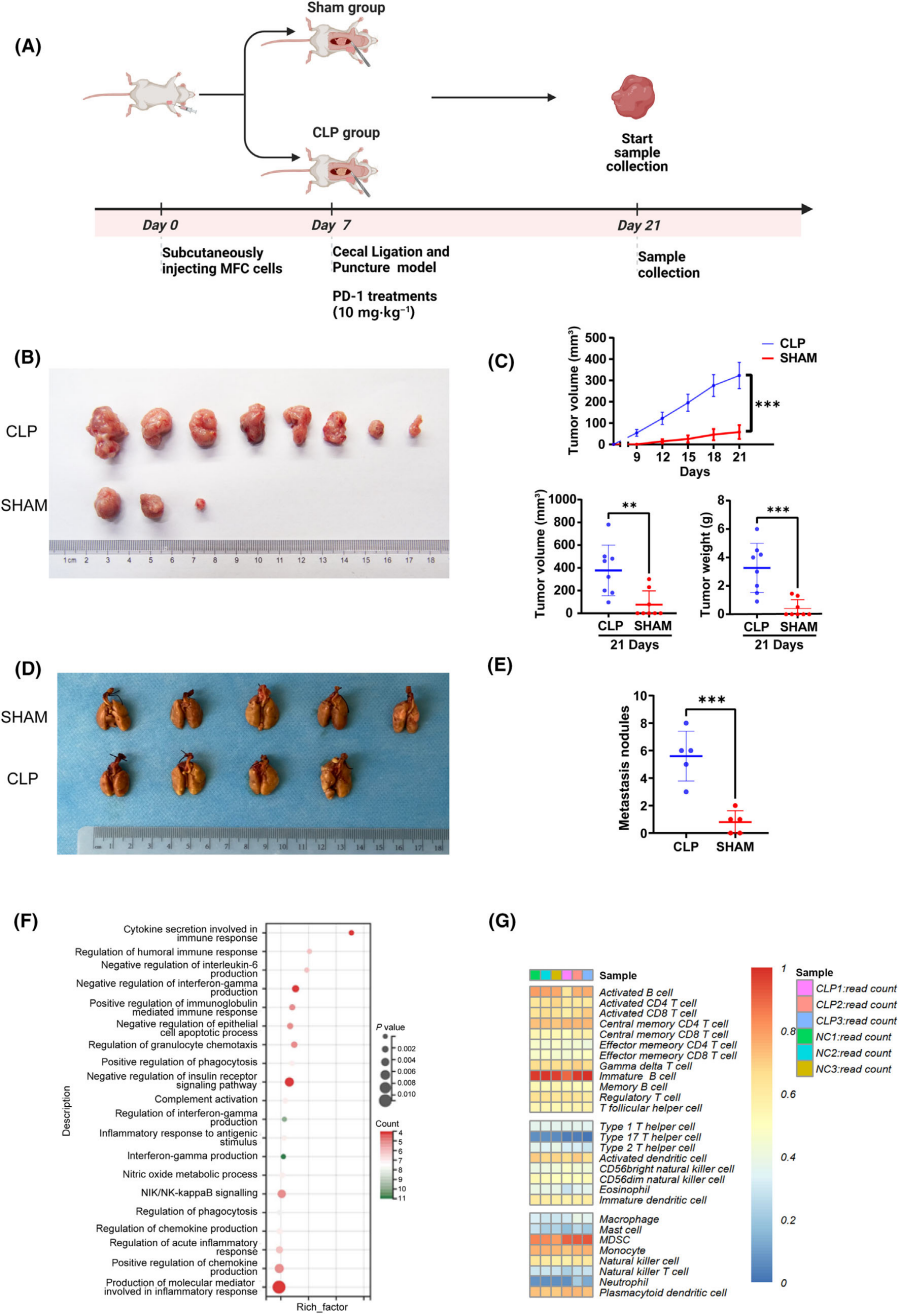
Abdominal infection promotes GC tumor growth and metastasis. (A) Schematic representation of gastric xenograft mouse model and CLP surgery. (B, C) The representative images and the quantification of xenograft upon MFC cell line injected 615 mice, respectively (*n* = 8 for each group). Tumor volumes were calculated after injection every 3 days for 21 days. Student's *t*‐test. (D, E) The representative images and the quantification of lung metastatic colonization of 615 mice treated with tail vein injection of MFC cells (*n* = 5 for each group). Student's *t*‐test. (F) KEGG pathway enrichment analysis was conducted to compare the differential gene expression between the CLP and SHAM groups in tumor samples. (G) CIBERSORT analysis was performed to assess immune cell infiltration in tumors following abdominal infection. Data are represented as mean ± SD. **P < 0.005, ***P < 0.001 versus the SHAM group by Student's *t*‐test. CLP, cecal ligation and puncture; GC, gastric cancer.

The effect of abdominal infection on tumor growth and metastasis was investigated through the implementation of a peritoneal metastasis model and a subcutaneous tumor model (Fig. 2A). The data revealed that the mean size of subcutaneously implanted tumors was significantly larger in the peritoneal infection group than in the control group (Fig. 2B,C). After tail vein implantation, the peritoneal infection group exhibited a significantly higher incidence of metastatic lung nodules than the control group (Fig. 2D,E).

To explore the causes of accelerated tumor growth following abdominal infection, we conducted RNA‐seq on tumor tissues obtained from tumor‐bearing mice. RNA‐seq analysis of tumors revealed that differentially expressed genes were significantly enriched in numerous immune regulatory pathways (Fig. 2F). Immune infiltration analysis revealed a significant increase in the expression of marker genes for MDSCs and neutrophils in tumors from the abdominal infection group (Fig. 2G).

### 3.3 The development of tumor‐immunosuppressive microenvironment accompanying with increased PD‐1 and decreased T cells level during abdominal infection

**Fig. 3:**
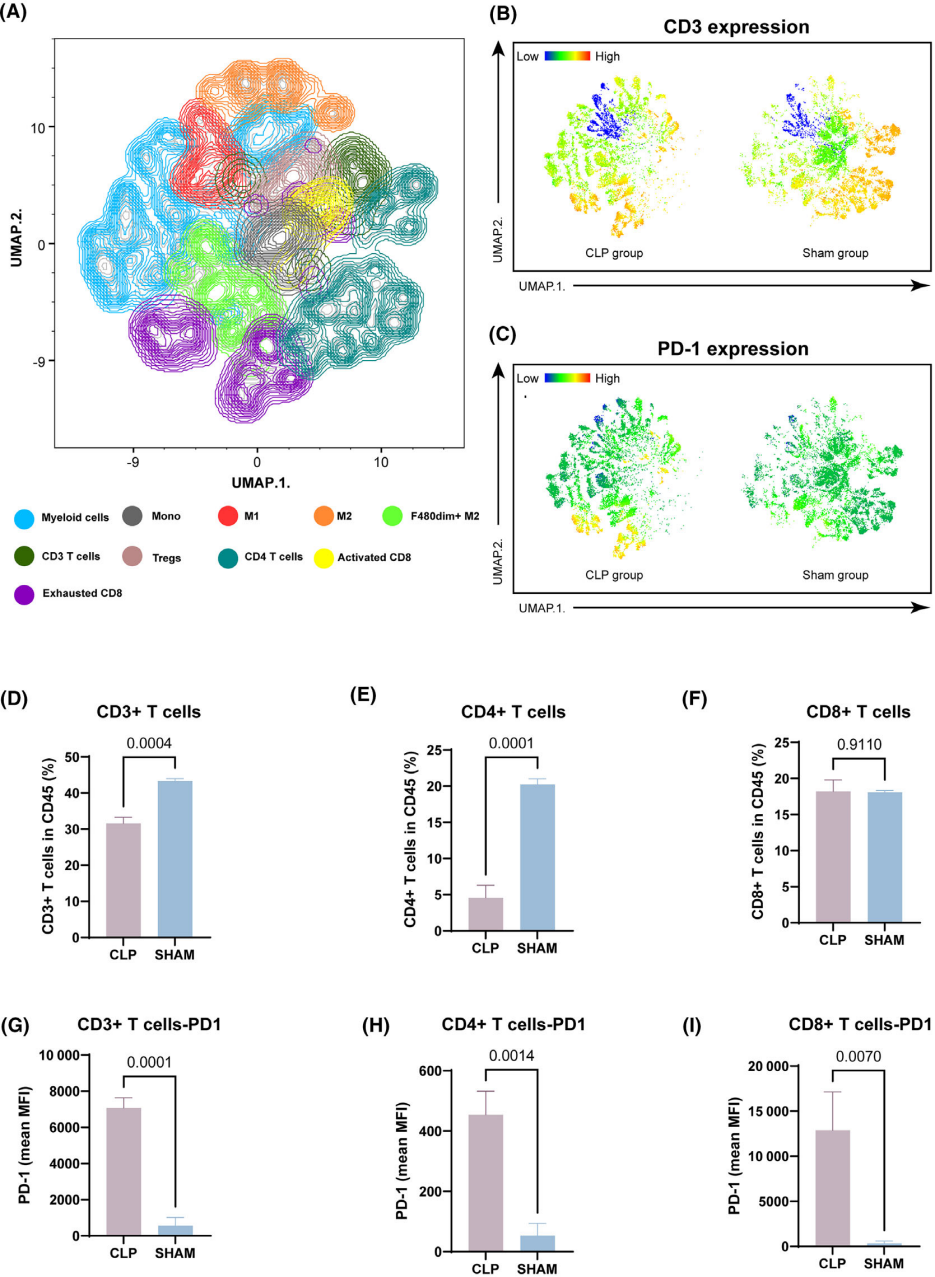
The development of a tumor‐immunosuppressive microenvironment is accompanied by an increase in PD‐1 expression and a decrease in T‐cell levels during abdominal infection. (A) UMAP projection of both phenotypic proteins. (B, C) Heatmap of UMAP projection of CD3 and PD‐1 expression in CLP and sham groups. (D–F) Changes in the percentage of CD3^+^, CD4^+^, and CD8^+^ T lymphocytes in tumors following abdominal infection. Student's *t*‐test, *n* = 3. (G–I) Changes in the expression intensity of PD‐1 on CD3^+^, CD4^+^, and CD8^+^ T lymphocytes in tumors following abdominal infection. Student's *t*‐test, *n* = 3. Data are represented as mean ± SD. CLP, cecal ligation and puncture; M1/M2, M1 macrophages and M2 macrophages; MFI, mean fluorescence intensity; Mono, monocyte; UMAP, uniform manifold approximation and projection.

To investigate the development of the tumor immune microenvironment following abdominal infection, 16‐parameter flow cytometry was employed to analyze single cell suspensions derived from tumors at 21 days post CLP (antibodies listed in Table S1). We pooled the flow cytometer‐acquired data on CD45^+^ immune cells of all samples in a single analysis with UMAP (Fig. 3A). SPADE is an unsupervised algorithm that aims to group cells into nodes that can be displayed on the UMAP. Compared to the sham group, there was a significant reduction in the number of CD45^+^ immune cells within the tumors of mice in the CLP group. UMAP projection heatmaps of CD3 and PD‐1 expression show loss of CD3 and upregulation of PD‐1 after abdominal infection in the CLP and sham groups (Fig. 3B,C). Similarly, a marked decrease was observed in the quantities of CD3^+^, CD4^+^, and PD1 negative CD8^+^ lymphocytes within tumors from mice in the CLP group (Fig. 3D–F). When targeting T cells, FACS analysis revealed that the surface expression of PD‐1 on CD4^+^ and CD8^+^ T cells within the tumor was upregulated at a heightened level after abdominal infection (Fig. 3G–I).

### 3.4 MDSC is enriched in tumors during abdominal infection and PDL1 is elevated

**Fig. 4:**
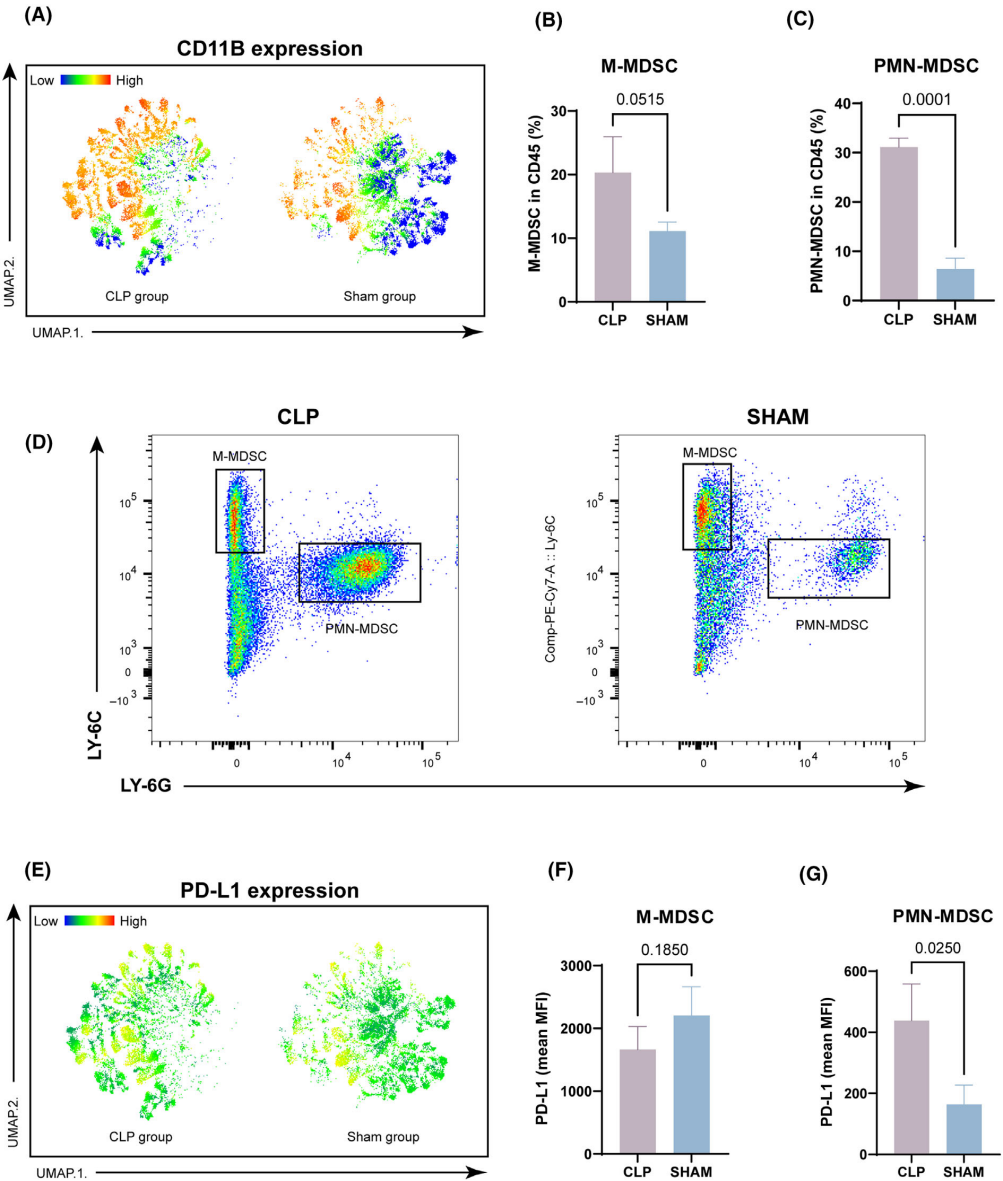
MDSCs are enriched in tumors during abdominal infection, accompanied by an elevation in PD‐L1 expression. (A) Heatmap of UMAP projection of CD11B expression in CLP and sham groups. (B–D) Changes in the percentage of monocytic (M‐MDSCs) and polymorphonuclear (PMN‐MDSCs) myeloid‐derived suppressor cells among CD45‐positive cells in tumors following abdominal infection. Student's *t*‐test, *n* = 3. (E) Heatmap of UMAP projection of PD‐L1 expression in CLP and sham groups. (F, G) The mean fluorescence intensity (MFI) of PD‐L1 expression in two types of MDSCs (M‐MDSC and PMN‐MDSC). Student's *t*‐test, *n* = 3. Data are represented as mean ± SD. CLP, cecal ligation and puncture; MDSCs, myeloid‐derived suppressor cells; MFI, mean fluorescence intensity; PMN, Polymorphonuclear; UMAP, uniform manifold approximation and projection.

In exploring the immunosuppressive microenvironment, we observed a notable rise in CD11B, a myeloid cell marker, within tumors post‐abdominal infection. MDSCs are pivotal in sepsis‐induced immunosuppression (Fig. 4A). MDSCs play a crucial role in mediating immunosuppression during sepsis, making their quantity a vital parameter to consider. To elucidate, the variation in quantity of MDSCs and the subsets was evaluated by the cells from tumor using flow cytometry. The two main subsets of MDSCs, PMN‐MDSCs and M‐MDSCs, were labeled as CD11b^+^Ly6G^+^Ly6C^low^ and CD11b^+^Ly6G^−^Ly6C^high^, respectively [30]. A dramatic increase was observed in MDSCs from the CLP group compared to the Sham group, with PMN‐MDSCs displaying a similar trend (Fig. 4B–D). PD‐L1, expressed on tumor and immune cells, triggers T‐cell apoptosis and immunosuppression via PD‐1 interaction. Flow cytometry revealed elevated PD‐L1 expression on MDSCs, particularly PMN‐MDSCs, in the CLP group, indicating their pivotal role in immune modulation post‐abdominal infection (Fig. 4E–G).

### 3.5 PMN‐MDSCs from mice with abdominal infection inhibits the proliferative capacity of T cells

**Fig. 5:**
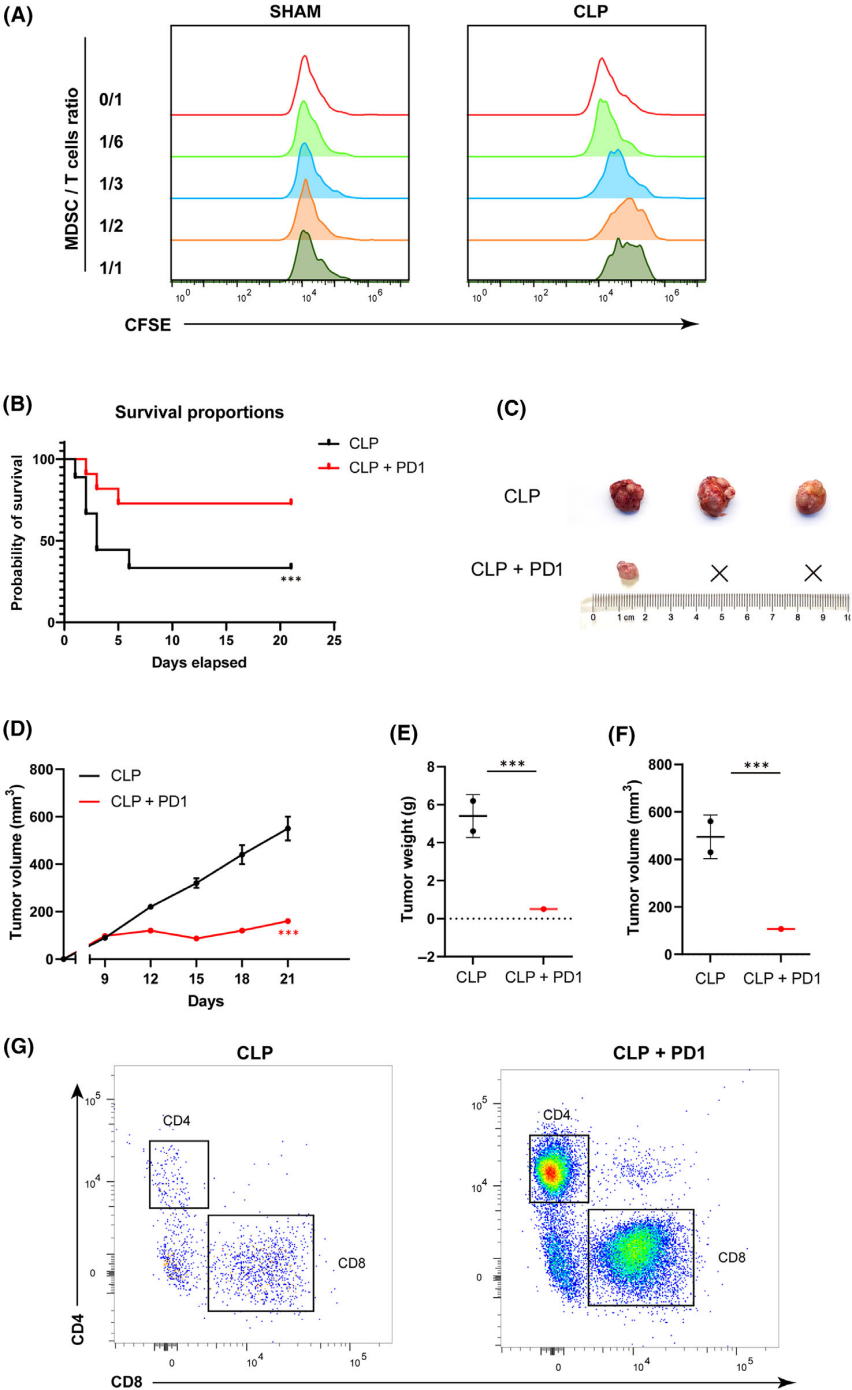
MDSCs are enriched in tumors during abdominal infection, accompanied by an elevation in PD‐L1 expression. (A) CD11b^+^ Ly6C^low^ Ly6G^+^ PMN‐MDSC, isolated by microbead‐assisted cell sorting, were co‐cultured with healthy CD3^+^T cells at ratios of 0/1, 1/1, 1/2, 1/3, and 1/6 (PMN‐MDSC/T). Representative histograms demonstrated the proliferation of activated T cells in the presence of PMN‐MDSC from Sham and CLP mice (*n* = 3 for each group). (B) Survival rate after surgery from day 1 to day 21. Data from each figure are summarized from three independent time‐course experiments. (C–F) Representative images and quantification results of xenografts treated with PD‐1 antibody in tumor‐bearing 615 mice with abdominal infection (three mice per group). Student's *t*‐test. (G) Changes in the percentage of CD4^+^ and CD8^+^ T lymphocytes among CD45 positive cells in tumors treated with PD‐1 antibody. Data are represented as mean ± SD of three independent experiments. ***P < 0.001 (compared with the SHAM group) by Student's *t*‐test. CLP, cecal ligation and puncture; MDSCs, myeloid‐derived suppressor cells; PMN, polymorphonuclear; UMAP, uniform manifold approximation and projection.

The findings indicated that PMN‐MDSC could be the primary group responsible for the inhibitory effect through PD‐L1. To assess the suppressive activity of post‐infection PMN‐MDSCs, tumor‐derived PMN‐MDSCs from both groups were isolated and co‐cultured with splenocytes from healthy mice, where T cells had been activated using anti‐CD3/CD28 microbeads. We observed that while PMN‐MDSCs from Sham mice exhibited some capacity to inhibit T‐cell proliferation at higher PMN‐MDSC proportions, PMN‐MDSCs derived from CLP mice demonstrated a significantly stronger inhibitory effect in a ratio‐dependent manner. Notably, the difference between the two groups was significant when the cell ratio was 1 : 1 (Fig. 5A).

### 3.6 Anti‐PD‐1 therapy can enhance the survival rate of mice with abdominal infection and restore immune function

Given the reciprocal relationship between PD‐1 and PD‐L1 expression and neutrophil, monocyte, CD8 T‐cell, and NK‐cell function, we hypothesized that the functional deficit could be restored by both anti‐PD‐1‐ and anti‐PD‐L1‐specific mAbs. Indeed, we observed that PD1 antibody significantly reduced the mortality rate of CLP mice (Fig. 5B). More than half of CLP mice died within 5 days, and only 36.4% of CLP mice survived till day 21 (Fig. 5B). After PD1 treatment, 81.8% of CLP survived within 2 days and could maintain a high survival rate (72.7%) up to 21 days. Moreover, treatment with PD‐1 antibody significantly elevated the proportions of CD4 and CD8 lymphocytes in the tumors of CLP mice, restoring them to levels akin to those observed in the sham group (Fig. 5G). Simultaneously, treatment with PD‐1 antibody significantly reduced the tumor engraftment rate and tumor growth rate in the abdominal infection group (Fig. 5C–F).

## 4 Discussion

The relationship between abdominal infection and long‐term survival in GC patients has been extensively studied, but the results remain controversial [6, 31, 32]. Multiple studies have highlighted a link between postoperative infectious complications and unfavorable long‐term outcomes in patients with GC [31, 33]. Our abdominal infection model suggests that sustained MDSC enrichment contributes to sepsis‐induced tumor immune suppression via the PD‐L1/PD‐1 axis, explaining tumor recurrence and metastasis in patients with PIICs. PD‐1 antibody therapy significantly inhibits sepsis‐induced tumor recurrence *in vivo*.

The CLP procedure is the most commonly employed mouse model for investigating the development of PIIC [34], and it can effectively mimic postoperative PIIC in human patients. Therefore, the CLP model is commonly employed in studies investigating the pathogenesis of infection due to its widespread recognition for relevance and reproducibility in research. The survival rate observed in our study was 37.5% after 7 days, aligning with previous studies where the survival rate for mid‐grade sepsis mice was approximately 40% [23]. According to Sepsis 3.0, human sepsis is characterized by dangerous organ failure resulting from an abnormal immune response to infection [34]. Therefore, an infection is a critical factor in the onset and advancement of sepsis. Our research revealed that at least two organs, including the heart and liver, were damaged after CLP, validating that the model corresponds with the updated sepsis criteria. Additionally, we observed significant splenomegaly over time, an important indicator of immune response in CLP mice, highlighting the need for careful consideration of immune cell activation.

The mechanism underlying the impact of abdominal infection on the long‐term survival of patients is multifaceted. Previous studies suggest that during radical GC surgery, viable cancer cells may inadvertently spill into the peritoneal cavity, heightening the risk of recurrence [35, 36]. Ordinarily, individual disseminated cancer cells undergo apoptosis naturally within the body, and immune surveillance mechanisms effectively clear most of these cells [37]. However, the immune alterations triggered by abdominal infection may create a conducive environment that fosters tumor recurrence and metastasis.

Abdominal infections, especially sepsis, result in a surge of pro‐ and anti‐inflammatory cytokines, leading to chronic immunoparalysis characterized by impaired CD4 and CD8 αβ T cell responses in the post‐septic environment [24]. The immune status in the blood is notably suppressed on the initial day of abdominal infection, marked by a comprehensive decrease in lymphocytes, neutrophils, and monocytes. Along those lines, we found an increase in CD11B^+^ myeloid cells in the abdominal infection group. At the same time, the most increased proportion is the PMN‐MDSC population, supporting the hypothesis of immune impairment in these patients. Several researchers have reported that MDSCs in patients with sepsis significantly elevate after the onset of abdominal infections [24]. Meanwhile, we showed that PMN‐MDSC with elevated PD‐L1 expression following CLP displayed immunosuppressive characteristics, restricting the proliferation of T cells in mice with sepsis. This study offers the first insight into how immune deterioration following abdominal infection influences the MDSC population in the tumor microenvironment, crucial for inducing tumor immune exhaustion.

Several limitations warrant consideration when interpreting the current study's findings, including the limitations of the experimental model and the long‐term effects and safety of treatment. The mouse model used in our study, while valuable for simulating certain pathological conditions observed in humans, may not fully recapitulate the complexity of the human condition. There are inherent differences between murine and human immune systems, which could affect the translation of our findings to clinical settings. We refrained from administering antibiotics to allow the infection to progress naturally, mirroring a clinical scenario. This approach offers a more accurate representation of the host's immune response to intra‐abdominal infection, which is a critical focus of our study, and better simulates the clinical setting. Additionally, elucidating the mechanisms underlying PIICs‐induced recurrence necessitates further exploration of alterations in the immune microenvironment within the human abdominal cavity.

Previous studies have demonstrated that PD‐1 deficiency or administration of anti‐PD‐1 antibodies can improve survival rates in mouse models of sepsis [36]. Our study reveals that PD‐1 blockade significantly enhances CLP mice's survival, accompanied by a marked increase in CD4^+^ and CD8^+^ T cells. This suggests that the beneficial effects of PD‐1 blockade may stem from the restoration of T‐cell function. Furthermore, we included a sham control group, where mice underwent subcutaneous tumor implantation without CLP surgery (Fig. 1A). The 100% survival rate in the sham group suggests that the observed mortality may not be solely attributed to tumor growth effects. Additionally, the restoration of T‐cell functionality, including cell proliferation and apoptosis, could potentially contribute to the elevated survival rate [38]. Thus, PD‐1 blockade may have a dual therapeutic benefit for postoperative GC patients with infections‐both inhibiting tumor growth and mitigating infection‐related damage through the enhancement of T‐cell responses.

Surgery remains crucial for solid tumor treatment, yet postoperative infectious complications are common. Our study underscores the need to prevent these complications, especially in locally advanced GC, and elucidates how they contribute to metastasis. The advent of immune checkpoint inhibitors has revolutionized cancer therapy, overcoming previous treatment limitations. However, identifying the patient populations that would derive the most benefit from such therapies remains an ongoing area of research [39]. Our research confirms that anti‐PD‐1 antibodies can prevent sepsis‐induced lymphocyte depletion and improve survival. These results highlight the therapeutic potential of PD‐1 inhibition in cancer management and mitigating infection‐related immune dysfunction, offering a dual benefit in patients with sepsis or postoperative infections. Simultaneously, the development of potential therapeutic approaches, such as target‐recruiting molecules inhibiting MDSC accumulation in tumors, should have profound implications for cancer patients experiencing surgery‐associated infections.

## 5 Conclusion

Our findings demonstrate that sepsis, simulated via a murine CLP model, contributes to an immunosuppressive tumor microenvironment by promoting T‐cell exhaustion and an increased presence of MDSCs expressing PD‐L1. This immune dysregulation supports cancer cell proliferation and metastatic potential. Treatment with anti‐PD‐1 antibodies effectively restored T‐cell function and reduced both mortality and metastasis in CLP‐induced septic mice. These results suggest that targeting the PD‐1/PD‐L1 pathway could be a promising therapeutic strategy for managing sepsis‐associated immune dysfunction in cancer patients.

## Conflict of interest

The authors declare no conflict of interest.

## Author contributions

YW and ZW was involved with the study concept and design, acquisition of data, drafting of the manuscript. TG and XX was involved with basic study concept and design. XL was involved with flow cytometry. XG performed bioinformatics analysis, while YL and FS provided assistance in experimental design. JJ and ZL was involved with the study concept and design and obtained funding, technical support, and study supervision. All authors read and approved the final manuscript.

### Peer review

The peer review history for this article is available at https://www.webofscience.com/api/gateway/wos/peer‐review/10.1002/1878‐0261.13767.

## Supporting information


**Fig. S1.** Organ injuries were evaluated using biomarkers (ALT, AST, DBIL, ALB, CK, LDH) post‐surgery.

**Table S1.** Design of flow cytometry antibody panel.

## Data Availability

Individual participant data and other data supporting the findings of this study are available from the corresponding authors on reasonable request.
