# Supplementary figures and images for: The accumulation of myeloid‐derived suppressor cells participates in abdominal infection‐induced tumor progression through the PD‐L1/PD‐1 axis
Source: Mol Oncol. 2025 Jan 21;19(5):1532–45. doi: 10.1002/1878-0261.13767 (PMC12077272; doi:10.1002/1878-0261.13767)

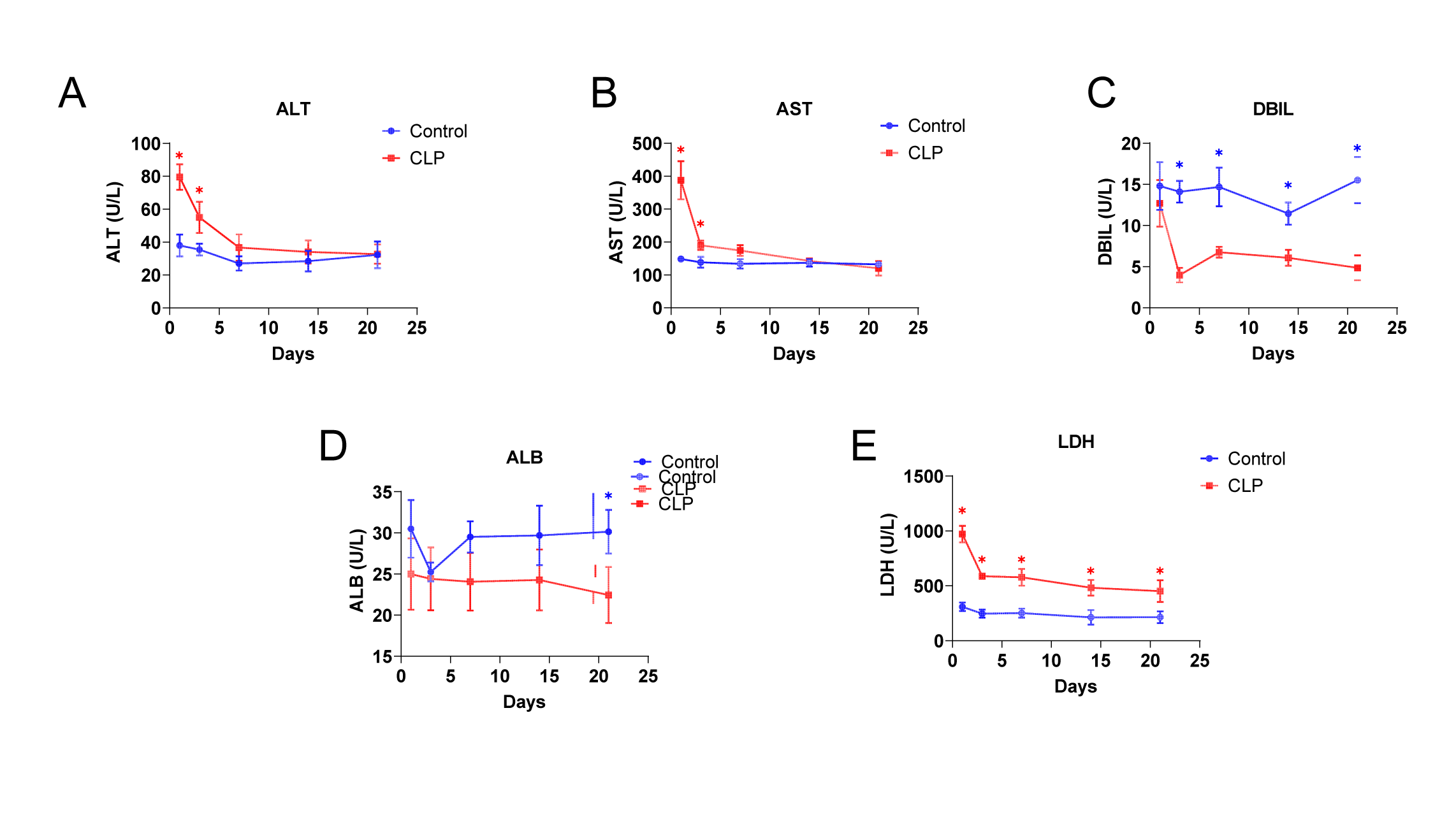

Supplement: Supplementary file 1 — Fig. S1. Organ injuries were evaluated using biomarkers (ALT, AST, DBIL, ALB, CK, LDH) post‐surgery. [file MOL2-19-1532-s001.tif]
