# Supplementary material for: The accumulation of myeloid‐derived suppressor cells participates in abdominal infection‐induced tumor progression through the PD‐L1/PD‐1 axis
Source: Mol Oncol. 2025 Jan 21;19(5):1532–45. doi: 10.1002/1878-0261.13767 (PMC12077272; doi:10.1002/1878-0261.13767)
Supplement: Supplementary file 2 — Table S1. Design of flow cytometry antibody panel. [file MOL2-19-1532-s002.docx]

Figure S1. Organ injuries were evaluated using biomarkers (ALT, AST, DBIL, ALB, CK, LDH) post-surgery.

Table S1:

| Panel 1: Insights into the Immune Microenvironment |  |
| --- | --- |
| **Fluor** | **Marker** |
| BB515 | CD11b |
| PerCp-Cy5.5 | CD3 |
| PE | F4/80 |
| PE-Cy7 | Ly-6C |
| PE-CF594 | PD-1 |
| APC | CD206 |
| R718 | CD326 |
| APC-Cy7 | L/D |
| BV421 | PD-L1 |
| BV510 | CD45 |
| BV605 | CD8 |
| BV650 | CD4 |
| BV711 | CD86 |
| BV786 | CD25 |
|  |  |
| Panel 2: Myeloid-Derived Suppressor Cells (MDSC) Insights |  |
| **Fluor** | **Marker** |
| BB515 | CD11b |
| PE-Cy7 | Ly-6C |
| PE-CF594 | PD-1 |
| APC | Ly-6G |
| APC-Cy7 | L/D |
| BV421 | PD-L1 |
| BV510 | CD45 |
